# Evidence for Distinct Mechanisms in the Shaping of the CD4 T Cell Repertoire in Histologically Distinct Myasthenia Gravis – Associated Thymomas

**DOI:** 10.1155/2001/49127

**Published:** 2001

**Authors:** P. Ströbel, M. Helmreich, H. Kalbacher, H. K. Müller-Hermelink, A. Marx

**Affiliations:** 1 Department of Pathology Pathologisches Institut University of Würzburg Josef-Schneider-Strasse 2 Würzburg D-97080 Germany; 2 Institute of Physiological Chemistry Tübingen Germany

**Keywords:** Cortical, epithelium, medullary, MHC class II, Myasthenia gravis, Thymoma, Thymus

## Abstract

The major histocompatibility complex (MHC) class II is involved both in thymocyte maturation and peptide presentation and might thus play a key role in the pathogenesis of paraneoplastic myasthenia gravis (MG) in thymomas. To further investigate this issue, we analyzed and scored the expression of epithelial class II expression in 35 thymomas (medullary, MDT; mixed, MXT; cortical and well differentiated thymic carcinoma, CT / WDTC) and correlated it with the histological tumor subtype, prevalence of MG and thymocyte maturation, which was analyzed by flow cytometry and RT-PCR. Our results show that both MHC class II expression and thymocyte maturation are highly dependent on the histological tumor subtype. CT / WDTC retain features of the normal outer thymic cortex, namely substantial MHC class II expression together with normal early thymocyte maturation until late phases of positive selection, but disturbed terminal thymopoiesis. By contrast, MDT and MXT retain features of the normal inner cortex and the medulla with low to absent class II expression and highly abnormal early thymocyte maturation including impaired positive selection, while terminal T cell maturation in MXT appeared undisturbed. There was no correlation between MHC class II expression and MG status for a given tumor subtype. In conclusion, our results provide evidence for a different histogenesis of cortical thymomas and well differentiated carcinomas on the one hand and mixed and medullary thymomas on the other.

Decreased expression levels of MHC class II, although of crucial importance for abnormal intratumorous maturation, are not sufficient to explain the emergence of paraneoplastic MG.


*Developmental Immunology*, 2001, Vol. 8(3-4), pp. 279-290
Reprints available directly from the publisher
Photocopying permitted by license only

© 2001 OPA (Overseas Publishers Association)
N.V. Published by license under
the Harwood Academic Publishers imprint,
part of The Gordon and Breach Publishing Group,
a member of the Taylor and Francis Group

### Evidence for Distinct Mechanisms in the Shaping of the CD4 T Cell Repertoire in Histologically Distinct Myasthenia Gravis – Associated Thymomas

P. STRÖBEL[a*], M. HELMREICH[a], H. KALBACHER[b], H.K. MÜLLER-HERMELINK[a] and A. MARX[a]

[a]*Department of Pathology, University of Würzburg, Josef-Schneider-Strasse 2, D-97080 Würzburg, Germany and* [b]*Institute of Physiological Chemistry, Tübingen, Germany*

The major histocompatibility complex (MHC) class II is involved both in thymocyte maturation and peptide presentation and might thus play a key role in the pathogenesis of paraneoplastic myasthenia gravis (MG) in thymomas. To further investigate this issue, we analyzed and scored the expression of epithelial class II expression in 35 thymomas (medullary, MDT; mixed, MXT; cortical and well differentiated thymic carcinoma, CT / WDTC) and correlated it with the histological tumor subtype, prevalence of MG and thymocyte maturation, which was analyzed by flow cytometry and RT-PCR. Our results show that both MHC class II expression and thymocyte maturation are highly dependent on the histological tumor subtype. CT / WDTC retain features of the normal outer thymic cortex, namely substantial MHC class II expression together with normal early thymocyte maturation until late phases of positive selection, but disturbed terminal thymopoiesis. By contrast, MDT and MXT retain features of the normal inner cortex and the medulla with low to absent class II expression and highly abnormal early thymocyte maturation including impaired positive selection, while terminal T cell maturation in MXT appeared undisturbed. There was no correlation between MHC class II expression and MG status for a given tumor subtype. In conclusion, our results provide evidence for a different histogenesis of cortical thymomas and well differentiated carcinomas on the one hand and mixed and medullary thymomas on the other.

Decreased expression levels of MHC class II, although of crucial importance for abnormal intratumorous maturation, are not sufficient to explain the emergence of paraneoplastic MG.

*Keywords:* Cortical, epithelium, medullary, MHC class II, Myasthenia gravis, Thymoma, Thymus

### INTRODUCTION

Myasthenia gravis (MG) is a neurological disorder characterized by autoantibodies against the acetylcholine receptors at the neuromuscular junction, resulting in generalized muscle weakness. The clinical presentation of MG is almost invariably associated with pathological alterations of the thymus (Marx et al. 1997). About 10 % of MG cases are related to thymic epithelial tumors (thymomas), which have been subdivided into medullary (MDT), mixed (MXT) and cortical thymomas (CT) and well differentiated thymic carcinomas (WDTC) based on the morphological resemblance of the neoplastic to normal thymic

* Corresponding author Dr. Philipp Ströbel, Pathologisches Institut, Universität Würzburg, Josef-Schneider-Strasse 2, D-97080 Würzburg, Germany. Phone: (49) 931 – 201 – 3878, Fax: (49) 931 – 201 – 3440, E-mail: path036@mail.uni-wuerzburg.de epithelium (Kirchner et al. 1992, Müller-Hermelink et al. 1994).

Thymomas characteristically retain organotypic features of the normal thymus, especially the capacity to generate mature T cells. Thus, a maintained but non-tolerogenic intratumorous thymopoiesis plus export of mature T cells from thymomas to peripheral lymphoid organs have been proposed to be a prerequisite for the development of paraneoplastic MG (Müller-Hermelink et al. 1997). However, although thymomas have been shown to be enriched in autoreactive T cells (Sommer et al. 1990; Nenninger 1998, Schultz 1999), the molecular basis of autoimmunization by thymomas remains largely enigmatic.

In recent years, some consistent features of these tumors have been described, including 1) intratumorous overexpression of autoantigen – related epitopes (Mygland et al. 1997; Wilisch et al. 1997, Schultz et al. 1999) and 2) impaired intratumorous thymopoiesis, particularly of the CD4 lineage (Takeuchi 1995; Nenninger 1997, 1998). Cells maturing in the thymus pass two critical checkpoints, positive and negative selection. During positive selection the thymic cortical epithelium presents an evolutionary optimized set of thymic peptides (Chan et al. 1993) bound to major histocompatibility complex (MHC) class I and II molecules to immature thymocytes. Depending on the MHC class recognized by the T cell receptor (TCR), either CD4 or CD8 are engaged and help to further increase the surface expression of the TCR and CD3 (Davis et al 1993). If the resulting avidity reaches a threshold level, the cell receives a survival signal. However, if the resulting avidity is too high, the cell is eliminated through apoptosis, a mechanism termed negative selection (Bevan 1997; Marrack and Kappler 1997; Williams et al. 1997; Jameson and Bevan 1998). Only cells surviving these two MHC-dependent checkpoints are allowed to complete maturation and leave the thymus (Janeway and Travers 1994). Although the MHC is thus critically involved in both thymocyte maturation and antigen presentation, it has not been clarified whether abnormal MHC expression levels are a consistent feature of thymomas (Willcox et al. 1987, Takeuchi et al. 1995, Nenninger et al. 1997) and whether alterations of MHC levels are related to abnormal thymopoiesis. Therefore we tried to further dissect the role of this molecule in intratumorous thymocyte maturation and in the pathogenesis of paraneoplastic MG.

### RESULTS

### Reduced Epithelial Expression of MHC Class II is a Consistent Feature of Thymomas, but Does Not Correlate with the Presence or Absence of Myasthenia Gravis

A reduced expression of epithelial HLA-DR at least in a subset of MG-associated thymomas has been described previously (van der Kwast 1985, Chilosi et al. 1986, Willcox et al. 1987, Takeuchi et al. 1995, Nenninger et al. 1997). However, there were no conclusive data on the prevalence and extent of this alteration and its significance for autoimmunity. In this study, we scored the expression of HLA-DR, DP, DQ and invariant chain (li) semiquantitatively (0 to 3 points for negative, weak, intermediate and strong staining intensity of each isotype), and calculated the cumulative MHC class II expression level (MHC-score) in a given case as the percentage of the maximal possible score.

Compared with adjacent thymic remnants and control thymuses, in which the average MHC-score was 88,5 %, class II expression was reduced in all instances regardless of the histological tumor subtype in a series of 35 thymoma cases. However, class II expression was lowest in those tumor types in which the prevalence of MG was lowest, namely medullary (average MHC-score 4 %, prevalence of MG 15,4 %) and mixed thymomas (average MHC-score 0,96 %, prevalence of MG 56 %). On the other hand, class II was significantly higher in those tumor types with high prevalence of MG, namely cortical thymomas and well differentiated thymic carcinomas (average MHC-score in cortical thymomas 21,2 %; prevalence of MG 75,8 %; average MHC-score in well differentiated thymic carcinomas 24,5 %; prevalence of MG 84,2 %) (Tab I). Within a thymoma subtype (e.g. among cortical thymomas), there was no correlation between the MHC-score and the presence or absence

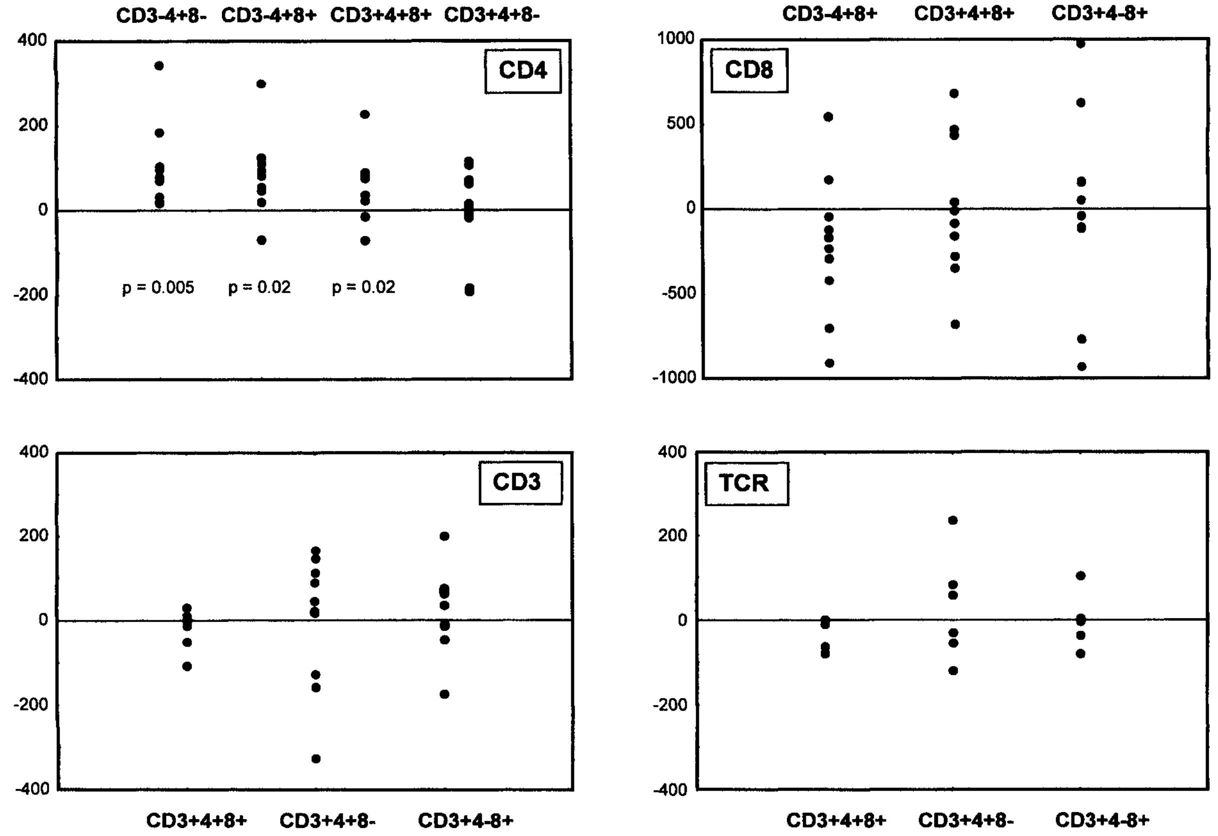

FIGURE 1 Intrapersonal comparison of median surface expression levels of CD4, CD8, CD3 and T cell receptor $\alpha\beta$ at different stages of thymocyte maturation in thymomas (flow cytometry). Dots indicate the difference between the value of the thymoma and the residual thymus of one individual patient. (4 mixed and 8 cortical thymomas / well differentiated carcinomas, 12 residual thymuses). As there were no statistically significant differences between mixed and cortical thymomas, all tumors were compared to the residual thymuses. In thymomas, surface CD4 was significantly increased on immature, pre-selective thymocytes, while there was no alteration in the surface expression of CD8, CD3 and the $\alpha\beta$ T cell receptor of MG. Of note, there were no differences in the staining intensity of MHC class I isotypes between thymomas and residual and control thymuses. These findings are in good agreement with earlier reports on reduced levels of MHC class II, but not class I, in thymoma epithelial cell lines (Papadopoulos et al. 1989).

TABLE I Histological subtypes and prevalence of myasthenia gravis in 164 thymoma cases and results of immunohistochemical analysis of MHC class II isotypes in 35 cases with available frozen material

| *Thymoma type* | *cases* | *MG+ (%)* | *IHC (MG+ / MG-)* | *MHC-II positive cases (MG+ / MG-)* |
|---|---|---|---|---|
| **medullary** | 13 | 2 (15,4) | 5 (1 / 4) | 1 (0 / 1) |
| **mixed** | 41 | 23 (56) | 8 (3 / 5) | 1 (1 / 0) |
| **cortical** | 91 | 69 (75,8) | 10 (7 / 3) | 5 (2 / 3) |
| **well differentiated thymic carcinomas** | 19 | 16 (84,2) | 12 (12 / 0) | 7 (7 / 0) |

MG: myasthenia gravis; IHC: cases available for immunohistochemical analysis of MHC class II isotypes, MHC-II positive cases (by IHC): MHC-score > 0 %.

### Immature, Pre-Selective T Cells In Thymomas Show Selective Upregulation of Surface CD4

CD4 plays a crucial role in positive selection by enhancing the intrinsically low avidity of the TCR for its ligands (Mc Carthy et al. 1988; Zuniga-Pflücker 1989; Dutz et al. 1995, Groves et al. 1997). In consequence, any alteration of either the surface expression of MHC or the TCR that would affect the TCR:MHC interaction would also require adjustment of the CD4 level in order to achieve a survival signal. Therefore, we compared the median expression levels of CD4, CD8, CD3 and the αβ T cell receptor on different thymocyte subsets in thymomas and residual thymuses by flow cytometry (Fig. 1).

Corresponding to the highly decreased expression of MHC class II on epithelial cells, thymoma-derived immature T cell populations of all histological tumor subtypes showed a significant upshift of surface CD4 compared to cells derived from residual and control thymuses. In particular, CD4 expression was significantly increased on CD3–4+8-, CD3–4+8+ and CD3+4+8+ cells, while this upshift was no longer detectable on mature CD3+4+8- cells. The MHC-class specifity of this alteration was underlined by the fact that CD8 surface expression did not show statistically significant alterations. Moreover, surface levels of CD3 and TCRαβ were virtually identical in thymomas and residual thymuses.

### The Shaping of the Mature CD4 T Cell Repertoire in Cortical Thymomas May Involve Other Factors than in Mixed Thymomas

It has been previously reported that thymocyte maturation in thymomas is quantitatively disturbed and that numbers of mature CD4 cells are significantly reduced, while the percentage of mature CD8 cells remains unremarkable (Takeuchi et al. 1995, Nenninger et al. 1997, Nenninger et al. 1998). As this constellation seemed to be related to the reduced MHC class II expression levels in thymomas, we tried to correlate our findings on subtype-specific reduction of class II with quantitative alterations in thymocyte maturation, which were assessed by three-colour flow cytometry. As the pathogenesis of MG in medullary thymomas has been considered to be linked to the particularly high numbers of recirculated T cells found in these tumors (Nenninger et al. 1997), they were not included in this study. In mixed thymomas, MHC class II expression was generally completely lost on the sensitivity level of immunohistochemistry. Although these tumors displayed an abundant production of immature CD3–4+8- and CD3–4+8+ precursor cells as reported previously (Takeuchi et al. 1995, Nenninger et al. 1997, Nenninger et al. 1998), most cells apparently were not able to enter into positive selection and were lost during transition into the CD3+4+8+ stage (Fig. 2). This finding is in good agreement with the particularly low levels of MHC class II in these tumors, as T cell development is known to be blocked at this stage also in the thymus of MHC I° II° mice (Chan et al. 1993, Grusby et al. 1993) and in patients with MHC class II deficiency (bare lymphocyte syndrome, BLS) (van Eggermond et al. 1993). However, the few cells which entered into positive selection seemed to complete maturation towards the pre-emigrant stage successfully. This suggests that positive selection might be the most important single determinant in the shaping of the mature thymocyte repertoire in mixed thymomas.

By contrast, cortical thymomas and well differentiated thymic carcinomas in many cases expressed substantial amounts of variable class II isotypes. Although positive selection was also impaired in CT / WDTC, it seemed to be better preserved and the numbers of CD69+ cells were significantly higher than in mixed thymomas. However, unlike in the normal thymus and also in mixed thymomas, a major proportion of these positively selected cells was eliminated before reaching the pre-emigrant CD3+4+8- stage, downsizing the frequency of CD3+4+8- cells to that of mixed thymomas. Considering that epithelial MHC class II is not required during terminal thymocyte maturation (Vanhecke et al. 1997), our findings suggest that factors independent of epithelial MHC class II play a major and qualitatively abnormal role in the shaping of the mature T cell repertoire in cortical thymomas and well differentiated carcinomas, but not in mixed thymomas as compared to normal thymus.

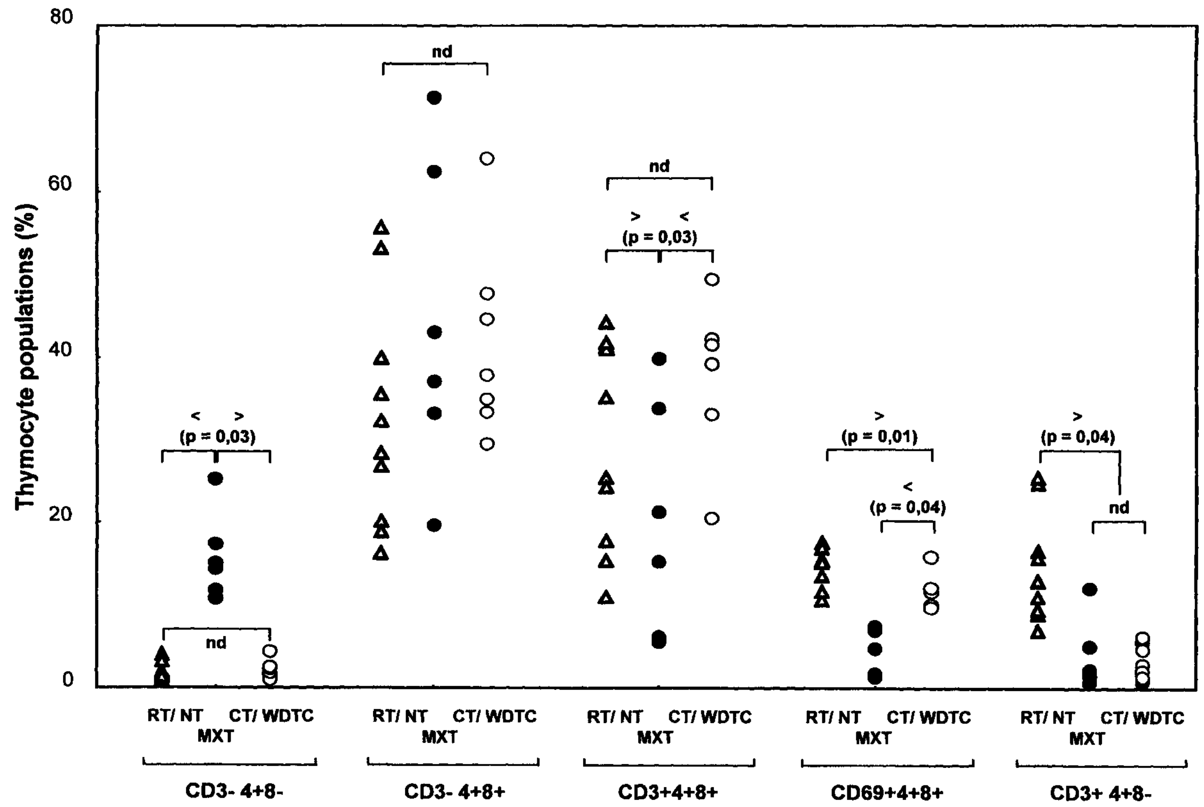

FIGURE 2 FACS analysis of main T cell subsets revealed significant differences in thymocyte maturation between mixed (MXT) and cortical thymomas / well differentiated carcinomas (CT / WDTC). In mixed thymomas, the majority of cells was lost before entry into positive selection, i.e. at the transition from the CD3–4+8+ to the CD3+4+8+ stage. However, the few positively selected (CD69+4+8+) cells in these tumors seemed to reach the mature CD3+4+8- stage. In cortical thymomas and well differentiated carcinomas, the numbers of positively selected cells were significantly decreased but still roughly comparable to residual and control thymuses (RT / NT). However, most of these cells were obviously eliminated before reaching the CD4$^{m}$ stage. Maturation of the CD8 lineage in all thymomas was undistinguishable from control thymuses (not shown). (nd: not statistically different)

### Reduced MHC Class II Expression in Thymomas Does Not Skew the T Cell Receptor Repertoire of Mature CD4 Cells

The interaction of the TCR with peptide / MHC class II complexes has been reported recently to play a predominant role in the shaping of the mature TCR repertoire (Sant'Angelo et al. 1997). To investigate the impact of reduced class II expression on the relative frequencies of TCR Vβ gene usage, we applied RT-PCR with a panel of 24 TCR Vβ family primers on highly purified, mature CD4 cells isolated from thymomas, residual thymuses and peripheral blood of the same patients. Medullary thymomas were excluded from this study for the same reasons as stated above. The overall TCR repertoire was not qualitatively affected neither in mixed nor in cortical thymomas and was not significantly different from residual thymuses and peripheral blood. Among the most prevalent families were TCR Vβ 5, 6, 8 and 13, all of which have been reported to be the predominant TCR Vβ segments also in normal (Baccala et al. 1991, Jores and Meo 1993) and non-neoplastic MG-associated thymuses (Truffault et al. 1997; Navaneetham et al. 1998). Minor, statistically non-significant alterations in the peripheral blood were attributed to confounding factors in this compartment, such as nonrandom homing to lymphatic tissues, different life spans etc. (Jores and Meo 1993). The analysis of CDR 3 length polymorphism did not reveal any differences between thymomas and residual and control thymuses. Thus, obviously neither intratumorous clonal deletions nor expansions do occur, as inferred from earlier studies based on TCR rearrangement studies (Katzin et al. 1988; Tesch et al. 1989; Scarpa et al. 1990).

### DISCUSSION

In this paper, we tried to assess the effects of reduced epithelial MHC class II on thymocyte maturation and the development of paraneoplastic myasthenia gravis. Our results show that reduced class II expression is a general and consistent feature of thymomas. However, both the extent of this reduction and its effects on thymocyte maturation appear to depend on the histological thymoma subtype.

Unlike in other tumors, where downregulation of MHC is a common finding with tumor progression (Vegh et al. 1993; Lefebvre et al. 1999), class II reduction was most pronounced in medullary and mixed thymomas, which show a biologically benign behaviour (Marx et al. 1999). Moreover, the selective downregulation of class II isotypes pointed to one or several defects in the pathways that regulate class II expression, such as INF-$\gamma$, STAT1 / IRF-1, or CIITA (Darnell 1997; Piskurich et al. 1999) or any other of the involved regulatory factors or, alternatively, overexpression of suppressive factors, such as TGF-$\beta$ (Piskurich et al. 1999). At the same time, medullary and mixed tumors show the lowest prevalence of myasthenia gravis among all organotypic thymomas, indicating that downregulation of MHC class II per se is not sufficient to explain the development of autoimmunity.

The effects of reduced class II expression on thymopoiesis seemed to be both qualitative and quantitative. Although maturation of the CD4 lineage was highly impaired, it was not altogether abrogated and thymomas always contained substantial numbers of phenotypically mature, pre-emigrant CD4 cells. This finding was further confirmed by the fact that the T cell receptor repertoire of highly purified pre-emigrant CD4 cells isolated from both mixed and cortical thymomas and well differentiated carcinomas displayed the same restriction pattern as T cells isolated from control thymuses.

In qualitative terms, upregulation of surface CD4 was equally observed in both mixed and cortical type thymomas and suggested that immature thymocytes indeed reacted to the altered microenvironment. While CD4 seems to play only a minor role in positive selection in the presence of wild-type peptide repertoires (Takahama et al. 1994a; van Bergen et al. 1998), it has great effects on the recognition of altered, suboptimal ligands (Vignali and Strominger 1994, Madrenas et al. 1997, van Bergen et al. 1998). Decreased MHC expression by itself may restrict the wild-type self-peptide repertoire, as only dominant peptide:MHC complexes are displayed, while minor complexes fall below the threshold level required for positive selection (Grubin et al. 1997) (Fig. 3). This effect is the more important, as the self-peptide repertoire in thymomas is most probably biased and not identical with the wild type repertoire (Mygland et al. 1997; Wilisch et al. 1997, Schultz et al. 1999). Upregulation of CD4 on maturing thymoctes in thymomas might lead to the irregular positive selection of autoreactive precursors that would otherwise not have received a survival signal. As the affinity required for positive selection lies threefold below the affinity for a signal inducing negative selection (Alam et al. 1996), it is conceivable that positively selected, autoreactive T cells might escape thymic deletion (Fig. 4).

In quantitative terms, reduction of class II expression corresponded with marked numerical alterations of thymocyte subpopulations, again with subtype-specifc variations.

In mixed thymomas, the observed subtotal loss of epithelial class II expression corresponded with a strong reduction of thymocyte numbers between the CD3–4+8+ and the CD4+8+69+ stage, indicating impaired intitiation of positive selection in this tumor type. Indeed, the capacity to induce positive selection has been ascribed uniquely to MHC class II+ thymic epithelial cells (Anderson et al. 1994 and 1999; Ernst

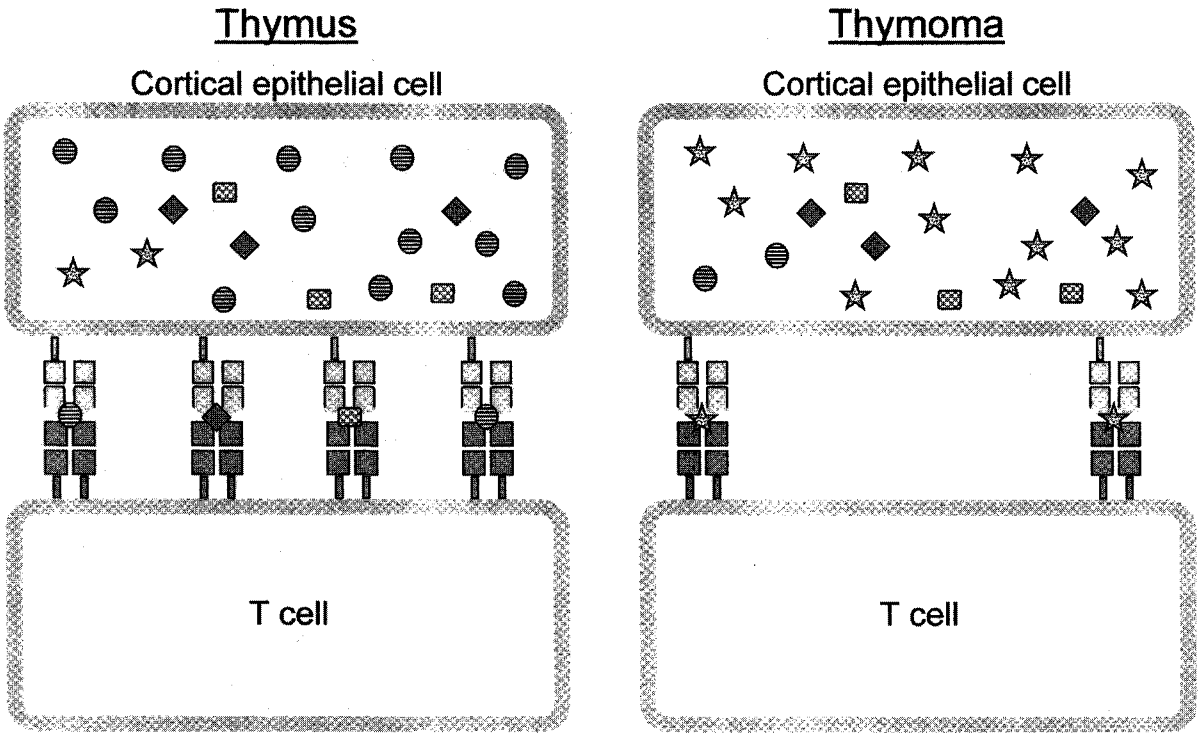

FIGURE 3 Hypothetical impact of reduced epithelial class II expression on peptide presentation: only frequent peptides are displayed, while minor peptides fall below the threshold level required for positive selection (Grubin et al. 1997). Moreover, due to overexpression of tumor-related antigens, the peptide repertoire presented by epithelial cells in thymomas is most probably biased and not identical with the wild-type repertoire et al. 1996). However, MHC-independent factors, such as TGF-β, have also been reported to be rate-limiting in the maturation of CD4+CD8+ thymocytes in both mice (Takahama et al. 1994b, Plum et al. 1995) and humans (Mossalayi et al. 1995). Interestingly, TGF-β has also been found to be involved in negative regulation of MHC class II (Lee et al. 1997; Nandan et al. 1997). However, the few cells that reached the CD4+8+69+ stage in mixed thymomas seemed to complete maturation successfully, suggesting that positive selection might be the most important single determinant in the shaping of the mature thymocyte repertoire in mixed thymomas.

By contrast, in line with the generally higher MHC class II levels observed in cortical thymomas and well differentiated thymic carcinomas, positive selection seemed to be better preserved in these tumors and the numbers of CD69+ cells were significantly higher than in mixed thymomas. However, unlike in the normal thymus and also in mixed thymomas, a major proportion of these positively selected cells was eliminated before reaching the pre-emigrant CD3+4+8- stage, downsizing the frequency of CD3+4+8- cells to that of mixed thymomas. We conclude from these findings that the initiation of positive selection in thymomas is crucially dependent on the expression of epithelial MHC class II. Although terminal maturation seems to be possible in the absence of MHC (Vanhecke et al. 1997), functional maturation still requires an intact thymic microenvironment (Dyall and Nikolic-Zigic 1995). Our findings thus suggest that factors independent of epithelial MHC class II play a major and qualitatively abnormal role in the shaping of the mature T cell repertoire in cortical thy-

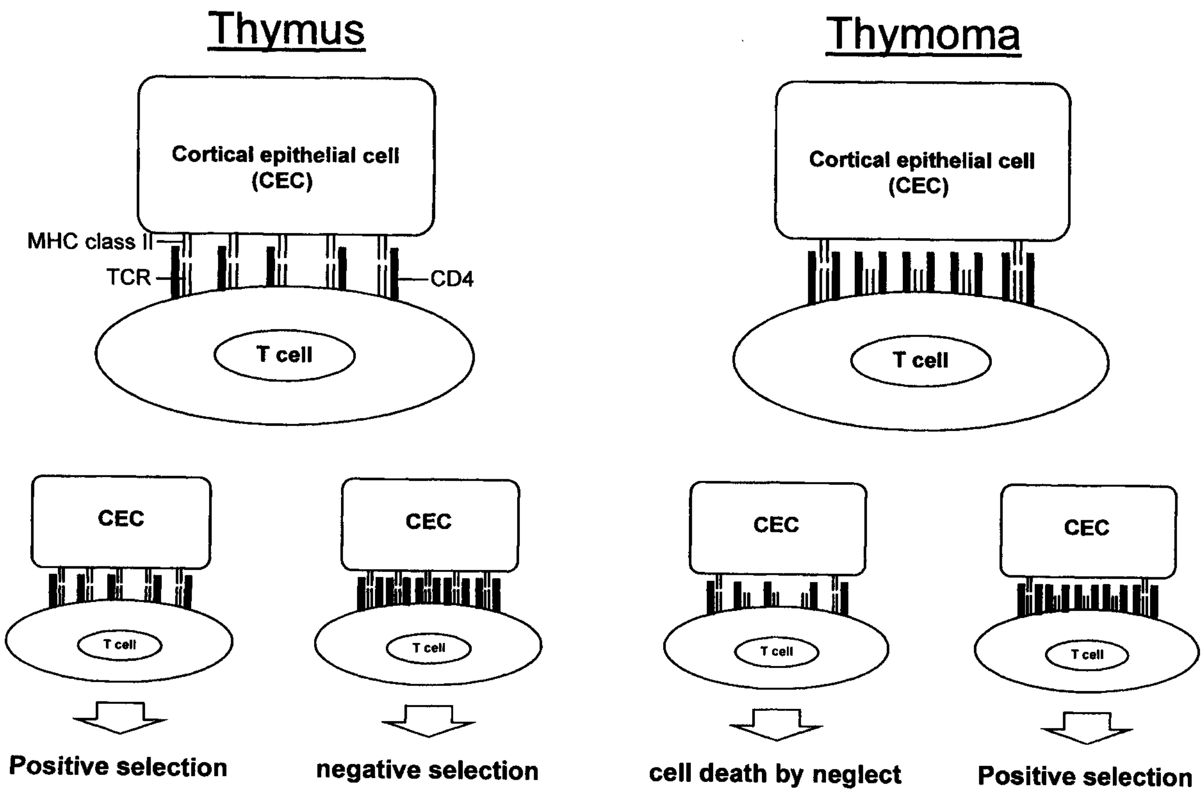

FIGURE 4 Potential selection advantage for pre-selection thymocytes with increased surface CD4 levels in thymomas with decreased MHC class II expression. In a micromilieu with low epithelial class II levels, upregulation of CD4 could convey a positively selecting signal, which under normal conditions would lead to the elimination (negative selection) of the cell. As the affinity required for positive selection is well below the affinity inducing negative selection, positively selected, autoreactive T cells might escape thymic deletion. Of note, CD4 upregulation was measured as median surface level and thus only a subset in a given thymocyte population in thymomas showed this alteration momas and well differentiated carcinomas, but probably not in mixed thymomas as compared to normal thymus. Interestingly, we have previously reported on an relative stromal overexpression of B7, a costimulatory molecule involved in negative selection (Kishimoto et al. 1996; Amsen and Kruisbeek 1996), in cortical thymomas (Marx et al. 1994). Other factors influencing the shaping of the CD4 lineage at the post-selection stage could be the 1) action of dendritic cells (Robey and Fowlkes 1994), 2) lack of MHC-independent survival signals such as LFA 1 and 3 / ICAM (Jorgensen et al. 1996), or VCAM (Salomon et al. 1997), or 3) abnormally accelerated export of mature T cells into the peripheral compartment.

In summary, both MHC class II expression and thymocyte maturation are highly dependent on the histological tumor subtype. CT / WDTC retain features of the normal outer cortical epithelium, namely substantial MHC class II expression together with unremarkable thymocyte maturation until late phases of positive selection. By contrast, MDT and MXT show characteristics of the inner cortical / medullary epithelium with low to absent class II expression (von Gaudecker et al. 1986) and highly abnormal early thymocyte maturation including impaired positive selection, while terminal T cell maturation appears undisturbed.

Our results thus provide further evidence for a different histogenesis of cortical thymomas and well differentiated carcinomas on the one hand and mixed and medullary thymomas on the other, as previously inferred from morphological observations (Müller-Hermelink et al. 1994).

While MHC class II expression and initiation of positive selection may be the central factors in the shaping of the T cell repertoire in mixed thymomas, positive selection is better preserved in cortical thymomas and well differentiated thymic carcinomas and additional, MHC-independent mechanisms seem to be predominant in these tumors. However, even heavy disturbances of thymocyte maturation and selection do not involve the T cell receptor repertoire of the few resulting pre-emigrant CD4 cells, at least as far as Vβ gene usage and CDR3 lenght polymorphism are concerned. Our failure to significantly correlate MHC II levels with MG status suggests that reduced epithelial MHC II expression by itself is not sufficient to explain the emergence of paraneoplastic myasthenia gravis.

### MATERIALS & METHODS

### Patients and Tumors

The tumors were subtyped according to the classification of Müller-Hermelink and coworkers (Kirchner et al. 1992; Müller-Hermelink et al. 1997). Cortical thymomas (CT) and well differentiated thymic carcinomas (WDTC), though morphologically distinct, are closely related in functional terms (Nenninger et al. 1997, 1998; Schultz et al. 1999). Therefore, they were considered as one group (CT / WDTC) to be compared with mixed thymomas (MXT). The diagnosis of MG was based on clinical findings and the detection of anti-AChR autoantibodies in the patient's serum.

### Immunohistochemical Detection and Semiquantitative Scoring of MHC Class II Isotypes

MHC class II epitopes were detected on frozen sections by a three-step immunoperoxidase technique as described in detail previously (Kirchner et al. 1988). The primary antibodies used were: L243 recognizing a complex nonpolymorphic epitope on mature HLA-DR dimers (Shackelford et al. 1983), Tü36 recognizing a complex epitope on mature and immature (invariant chain (li)-associated) HLA-DR dimers and Tü22 recognizing a monomorphic determinant on HLA-DQ (Klohe et al. 1988). FA (B7/21) recognizes a complex epitope on HLA-DP dimers (Klohe et al. 1988) and SD3 253.74 recognizes a simple C-terminal epitope of the invariant chain (li) (amino acids 194–209) (Max et al. 1993). All primary antibodies provided by Dr. H. Kalbacher, Institute of Physiological Chemistry, Tübingen, Germany). Epithelial cells in thymomas and thymuses were identified using a monoclonal antibody against cytokeratin 19, which is known to be highly expressed in all subtypes of thymomas (Grommisch et al. 1997). Other MHC class II expressing cell types, such as B cells, makrophages and dendritic cells, were identified using monoclonal antibodies against CD22 (Dako, Hamburg, Germany), CD 16 (Dianova, Hamburg) and CD11c (KiM1) (Bachem Biochemica, Heidelberg, Germany), respectively. MHC class I expression was analyzed using an anti-human HLA A, B, C monoclonal antibody (Dako-HLA-ABC, Dako, Hamburg, Germany). Intensity of MHC class II staining was assessed using a semi-quantitative point score (0 to 3 points for negative, weak, intermediate and strong staining intensity, respectively). Staining intensity was expressed as percent of maximal possible score in a given case.

### Flow Cytometry

Lymphocytes were immunophenotyped by three-color FACS analysis on a Becton Dickinson FACScan flow cytometer using the following monoclonal antibodies: isotype controls, anti-CD3, anti-CD4 (Quantum red labeled) (Sigma, Deisenhofen, Germany), anti-CD45RO (unkonjugated), anti-CD25 (PE-labeled), anti-CD19 (FITC-labeled) (Dako, Hamburg, Germany), PE-conjugated F(ab'$^2$) fragment donkey-anti-mouse IgG, anti-HLA-DR (PE-labeled) (Dianova, Hamburg, Germany), anti-CD45RA (FITC-labeled) (Coulter Immunotech;

Hamburg, Germany) and anti-TCRαβ (FITC-labeled) (Becton Dickinson, Heidelberg, Germany).

### Semiquantitative RT- PCR

Freshly isolated $CD3^{+}4^{+}8^{-}$ cells from native thymomas, residual thymuses and peripheral blood were isolated by a combination of depletion/selection procedures using cell isolation columns (Camon Labor Service, Wiesbaden, Germany) and immunomagnetic Beads (Dynal, Hamburg, Germany). The purity of the isolated subsets was controlled by FACS staining and was generally ≥ 95 %. Total RNA was prepared from purified $CD4^{m}$ ($10^{6}$) cells using the RNeasy Kit (Quiagen, Hilden, Germany). After cDNA synthesis with oligo-dT primers and MMLV reverse transcriptase (Gibco, Eggenstein, Germany), 1/20 of the reaction was amplified using Taq polymerase (Amersham, Braunschweig, Germany) and sequence-specific primers. A semiquantitative PCR was performed by adjusting all cDNAs to equal amounts of GAPDH transcripts. The T cell receptor Vβ (TCR Vβ) repertoire of a given sample was investigated using a panel of 24 TCR Vβ-specific primers and a fluorescent TCR Cβ-specific consensus primer as described previously (Genevee et al. 1992).

Quantitation was performed through digitalization of ethidium bromide-stained agarose gels and subsequent analysis using ONE-Dscan software (Scanalytics, Billerica, USA). Usage of a single TCR Vβ segment was expressed as percentage of the total product amplified by all the Vβ primers.

### Evaluation of CDR3 Length Polymorphisms

Aliqouts of the run-off products were subjected to electrophoresis using an ABI 373A sequencer (Applied Biosystems, Weiterstadt, Germany) in the presence of fluorescent ABI Rox-2500 marker. Evaluation of the CDR3 length polymorphisms was performed using the GenScan software version 1.2.2.

### Statistical Procedures And Data Plotting

Analysis of the flow cytometric data and creation of histograms were performed using the Lysis II software (Becton Dickinson). The density of different surface molecules was determined by using the median value provided by the software.

For all statistical analyses, data were computed using the Statistica software (StatSoft, Tulsa, USA) and tested for significant differences using the Mann-Whitney-U test ($p$ value $< 0.05$, unless otherways specified).

In this article, thymomas were subtyped according to the classification proposed by Müller-Hermelink et al. The thymoma subtypes used here correspond to the new World Health Organization classification, which distinguishes type A thymomas (medullary), type AB thymomas (mixed), type B thymomas (subclassified as type B1, predominantly cortical; B2, cortical; and B3, well differentiated thymic carcinomas) and type C thymomas (thymic carcinomas) (Rosai and Sobin 1999).

### *References*

Alam S.M., Travers P.J. Wung J.L., Nasholds W., Redpath S., Redpath S., Jameson S.C. Gascoigne N.R. (1996). T-cell-receptor-affinity and thymocyte positive selection. Nature **381**: 616–620.

Amsen D., Kruisbeek A. (1996). CD28-B7 interactions function to co-stimulate clonal deletion of double-positive thymocytes. Int. Immunol. **8**: 1927–36.

Anderson G., Owen J.J:T., Moore N.C. and Jenkinson E.J. (1994). Thymic epithelial cells provide unique signals for positive selection of CD4+8+ thymocytes in vitro. J. Exp. Med. 179: 2027–2031.

Anderson G., Hare K.J. and Jenkinson E.J. (1999). Positive selection of thymocytes: the long and winding road. Immunol. Today **20**: 463–468.

Baccala R, Kono DH, Walker S, Balderas RS, Theofilopoulos AN. (1991) Genomically imposed and somatically modified human thymocyte V beta gene repertoires. Proc. Natl. Acad. Sci. USA **88**: 2908–2912.

Bevan M.J. (1997). In thymic selection, peptide diversity gives and takes away. Immunity **7**: 175–178.

Chan S.H., Cosgrove D., Waltzinger C., Benoist C., Mathis D. (1993). Another view of the selective model of thymocyte selection. Cell **73**: 225–236.

Chilosi M, lannucci A, Fiore Donati L, Tridente G, Pampanin M, Pizzolo G, Ritter M, Bofill M, Janossy G. (1986). Myasthenia gravis: immunohistological heterogeneity in microenvironmental organization of hyperplastic and neoplastic thymuses suggesting different mechanisms of tolerance breakdown. J. Neuroimmunol. **11**: 191–204.

Darnell J.E. Jr. (1997). STATs and gene regulation. Science **277**: 1630–1635.

Davis C.B., Killeen N., Crooks M.E., Raulet D., Littman D.R. (1993). Evidence for a stochastic mechanism in the differentiation of mature subsets of T lymphocytes. Cell **73**: 237–247.

Dietrich P.Y., Caignard A., Lim A., Chung V., Pico J.L., Pannetier C., Kourilsky P., Hercend T., Even J., Triebel F. (1994). In vivo T-cell clonal amplification at time of acute graft-versus-host disease. Blood **84**: 2815–2820.

Dutz J.P., Ong C.J., Marth J., Teh H.S. (1995). Distinct differentiative stages of CD4+CD8+ thymocyte development defined by the lack of coreceptor binding in positive selection. J. Immunol. **154**: 2588–2599.

Dyall R. and Nikolic-Zigic J. (1995). The majority of postselection CD4+ single-positive thymocytes requires the thymus to produce long-lived, functional T cells. J. Exp. Med. **181**: 235–245.

Ernst B., Surh C.D. and Sprent J. (1996). Bone marrow-derived cells fail to induce positive selection in thymus reaggregation cultures. J. Exp. Med. **183**: 1235–1240.

Genevee C., Diu A., Nierat J., Caignard A., Dietrich P.Y., Ferradini L., Roman Roman S., Triebel F., Hercend T. (1992). An experimentally validated panel of subfamily-specific oligonucleotide primers (V alpha 1-w29/V beta 1-w24) for the study of human T cell receptor variable V gene segment usage by polymerase chain reaction. Eur. J. Immunol. **22**: 1261–1269.

Grommisch K. Hofmann W.J.,Otto H.F., Willgeroth K., Moll R. (1997). Complex and differential cytokeratin profiles in thymomas and correlation with normal thymus. In Epithelial tumors of the thymus. Marx A. and Müller-Hermelink H.K., eds. Plenum Press, New York pp. 81–89.

Groves T., Parsons M., Miyamoto N.G., Guidos C.J. (1997). TCR engagement of CD4+CD8+ thymocytes in vitro induces early aspects of positive selection, but not apoptosis. J. Immunol. **158**: 65–75.

Grubin C.E., Kovats S., deRoos P., Rudensky A. Y. (1997). Deficient positive selection of CD4 T cells in mice displaying altered repertoires of MHC class II-bound self-peptides. Immunity **7**: 197–208.

Grusby M.J., Auchincloss Jr. H., Lee R., Johnson R.S., Spencer J.P., Zijlstra M., Jaenisch R., Papaioannou V.E., Glimcher L.H. (1993). Mice lacking major histocompatibility complex class I and class II molecules. Proc. Natl. Acad. Sci. USA **90**: 3913–3917.

Jameson S.C. and Bevan M.J. (1998). T-cell selection. Curr. Opin. Immunol. **10**: 214–219.

Janeway C.A. and Travers P. (1994). In: Anonymous Current Biology Ltd/Garland Publishing Inc. New York. pp 241–277.

Jores R. and Meo T. (1993). Few V gene segments dominate the T cell receptor beta-chain repertoire of the human thymus. J. Immunol. **151**: 6110–6122.

Jorgensen A., Nielsen M. Svejgaard A., Ledbetter J.A., Odum N., Ropke C. (1996). Human thymic epithelial cells present superantigens to T-cell lines and thymocytes. Exp. Clin. Immunogenet. **13**: 192–203.

Katzin W.E., Fishleder A.J., Linden M.D., Tubs R.R. (1988). Immunoglobulin and T-cell receptor genes in thymomas: genotypic evidence supporting the nonneoplastic nature of the lymphocytic component. Hum. Pathol. **19**: 323–8.

Kirchner T., Tzartos S., Hoppe F., Schalke B., Wekerle H., Müller-Hermelink H.K. (1988) Pathogenesis of myasthenia gravis. Acetylcholine receptor-related antigenic determinants in tumor-free thymuses and thymic epithelial tumors. Am. J. Pathol. **130**: 268–280.

Kirchner T., Schalke B., Buchwald J., Ritter M., Marx A., Müller-Hermelink H.K. (1992). Well-differentiated thymic carcinoma: An organotypical low-grade carcinoma with relationship to cortical thymoma. Am. J. Surg. Pathol. **16**: 1153–1169.

Kishimoto H., Cai Z., Brunmark A., Jackson M.R., Peterson P.A., Sprent J. (1996). Differing roles for B7 and intercellular adhesion molecule-1 in negative selection of thymocytes. J. Exp. Med. **184**: 531–7.

Klohe E.P., Watts R., Bahl M., Alber C., Yu W.Y., Anderson R., Silver J., Gregersen P.K., Karr R.W., Analysis of the molecular specificities of anti-class II monoclonal antibodies by using L cell transfectants expressing HLA class II molecules. J. Immunol. **141**: 2158–2164.

Lee Y.J., Han Y., Lu, H.T., Nguyen V., Qin H., Howe P.H., Hocevar B.A., Moss J.M., Ransohoff R.M., Benveniste E.N. (1997). TGF-beta suppresses IFN-gamma induction of class II MHC gene expression by inhibiting class II transactivator messenger RNA expression. J. Immunol. **158**: 2065–75.

Lefebvre S., Moreau P., Dausset J., Carosella E.D., Paul P. (1999). Downregulation of HLA class I gene transcription in choriocarcinoma cells is controlled by the proximal promoter element and can be reversed by CIITA. Placenta **20**: 293–301.

Madrenas J., Chau L.A., Smith J., Bluestone J.A., Germain R.N. (1997). The efficiency of CD4 recruitment to ligand-engaged TCR controls the agonist/partial agonist properties of peptide-MHC molecule ligands. J. Exp. Med. **185**: 219–229.

Marino M., Müller-Hermelink H.K. (1985). Thymoma and thymic carcinoma. Virchows Arch A **407**: 119–149.

Marrack P. and Kappler J. (1997). Positive selection of thymocytes bearing alpha beta T cell receptors. Curr. Opin. Immunol. **9**: 250–255.

Marx A., Schömig D., Jung A., Schultz A., Gattenlöhner S., Brabletz T., Kirchner T., Müller-Hermelink H.K. (1994). Distribution of molecules mediating thymocyte-stroma-interactions in human thymus, thymitis and thymic epithelial tumors. Thymus **23**: 83–93.

Marx A., Wilisch A., Schultz A., Gattenlöhner S, Nenninger R., Müller-Hermelink H.K. (1997). Pathogenesis of myasthenia gravis. **430**: 355–64.

Marx A. and Müller-Hermelink H.K. (1999). From basic immunobiology to the upcoming WHO-classification of tumors of the thymus. The second conference on biological and clinical aspects of thymic epithelial tumors and related recent developments. Pathol. Res. Pract. **195**: 555–63.

Max H., Halder T., Kropshofer H., Kalbus M., Muller C.A. and Kalbacher H. (1993) Characterization of peptides bound to extracellular and intracellular HLA-DR1 molecules. Hum. Immunol. **38**: 193–200.

Mc Carthy S.A., Kruisbeek A.M., Uppenkamp I.K., Sharrow S.O., Singer A. (1988). Engagement of the CD4 molecule influences cell surface expression of the T cell receptor on thymocytes. Nature **336**: 76–79.

Mossalayi M.D., Mentz F., Quaaz F., Dalloul A.H., Blanc C., Debré C., Ruscetti F.W. (1995) Early human thymocyte proliferation is regulated by an externally controlled autocrine transforming growth factor-β1-mechanism. Blood **85**: 3594 – 3601.

Müller-Hermelink H.K., Marx A., and Kirchner T. (1994). Advances in the diagnosis and classification of thymic epithelial tumors. In: Recent advances in histopathology. ed. Anthony and Mac Sween, Churchill Livingstone, Edinburgh, pp. 49–63.

Müller-Hermelink H.K., Wilisch A., Schultz A., Marx A. (1997). Characterization of the human thymic microenvironment: lymphoepithelial interaction in normal thymus and thymoma. Arch. Histol. Cytol. **60**: 9–28.

Mygland A., Kuwajima G., Mikoshiba K., Tysnes O.B., Aarli J.A., Gilhus N.E. (1995). Thymomas express epitopes shared by the ryanodine receptor. J. Neuroimmunol. **62**: 79–83.

Nandan D., Reiner NE. (1997). TGF-beta attenuates the class II transactivator and reveals an accessory pathway of IFN-gamma action. J. Immunol. **158**: 1095–101.

Navaneetham D., Penn A.S., Howard J.F. Jr., Conti-Fine B.M. (1998). TCR-Vbeta usage in the thymus and blood of myasthenia gravis patients. J. Autoimmun. **11**: 621–33.

Nenninger R., Schultz A., Vandekerckhove B., Hünig T., Schalke B., Müller-Hermelink H.K., Marx A. (1997). Abnormal T lymphocyte development in myasthenia gravis-associated thymomas In Epithelial Tumors of the thymus. ed. Marx and Müller-Hermelink, Plenum Press, New York pp. 165–177.

Nenninger R., Schultz A., Hoffacker V., Helmreich M., Wilisch A., Vandekerckhove B, Hünig T., Schalke B., Schneider C., Tzartos S., Kalbacher H., Müller-Hermelink H.K., Marx A. (1998). Abnormal thymocyte development and generation of autoreactive T cells in mixed and cortical thymomas. Lab. Invest. **78**: 743–753.

Pannetier C., Even J., and Kourilsky P.(1995). T-cell repertoire diversity and clonal expansions in normal and clinical samples. Immunol. Today **16**: 176–181.

Papadopoulos T., Kirchner T. Müller-Hermelink H.K. (1989) Primary cultures of human thymic epithelial tumors. Morphological and immunocytochemical characterization. Virch. Arch. B Cell. Pathol. Incl. Mol. Pathol. **56**: 363–70.

Piskurich J.F., Linhoff M.W., Wang Y., Ting J.P.-Y. (1999). Two distinct gamma-interferon-inducible promoters of the major histocompatibility complex class II transactivator gene are differentially regulated by STAT1, Interferon regulatory factor 1, and transforming growth factor β. Moll. Cell. Biol. **19**: 431–440.

Plum J., De-Smedt M., Leclercq G, Vandekerckhove B. (1995). Influence of TGF-beta on murine thymocyte development in fetal thymus organ culture. J. Immunol. **154**: 5789–98.

Robey E., Fowlkes B.J. (1994). Selective events in T cell development. Annu. Rev. Immunol. **12**: 675–705.

Rosai J., Sobin L.H. (1999). Histological typing of tumours of the thymus. World Health Organization. International Classification of Tumours. Heidelberg, Springer Verlag, pp. 1-65.

Salomon D.R., Cirsa L., Mojcik C.F., Ishii J.K., Klier G., Shevach E.M. (1997). Vascular cell adhesion molecule-1 is expressed by cortical thymic epithelial cells and mediates thymocyte adhesion. Implications for the function of alpha4beal (VLA4) integrin in T-cell development. Blood **89**: 2461–71.

Sant'Angelo D.B., Waterbury P.G., Cohen B.E., Martin W.D., Van Kaer L., Hayday A.C., Janeway C.A. Jr. (1997). The imprint of intrathymic self-peptides on the mature T cell receptor repertoire. Immunity **7**: 517–524.

Scarpa A., Chilosi M., Capelli P., Bonetti F., Menestrina F., Zamboni G., Pizzolo G., Palestro G., Fiore-Donati L., Tridente G. (1990) Expression and gene rearrangement of the T-cell receptor in human thymomas. Virchows Arch. B Cell. Pathol. Incl. Mol. Pathol. **58**: 235–9.

Shackelford D.A., Lampson L.A., Strominger J.L. (1983). Separation of three class II antigens from a homozygous human B cell line. J. Immunol. **130**: 289–296.

Schultz A., Hoffacker V., Wilisch A., Nix W., Gold R., Schalke B., Tzartos S., Müller-Hermelink K.H., Marx A. (1999). Neurofilament is an autoantigenic determinant in myasthenia gravis. Ann. Neurol. **46**: 167–75.

Sommer N., Willcox N., Harcourt C., Newsom-Davis J. (1990). Myasthenic thymus and thymoma are selectively enriched in acetylcholine receptor-reactive T cells. Ann. Neurol. **28**: 312–19.

Takahama Y., Suzuki H., Katz K.S., Grusby M.J., Singer A. (1994a). Positive selection of CD4+ T cells by TCR ligation without aggregation even in the absence of MHC. Nature **371**: 67–70.

Takahama Y., Letterio J.J., Suzuki H., Farr A.G., Singer A., (1994b) Early progression of thymocytes along the CD4 / CD8 developmental pathway is regulated by a subset of thymic epithelial cells expressing transforming growth factor beta. J. Exp. Med. **179**: 1495–506.

Takeuchi Y., Fujii Y., Okumura M., Inada K., Nakahara K., Matsuda H. (1995). Accumulation of immature CD3-CD4+CD8-single-positive cells that lack CD69 in epithelial cell tumors of the human thymus. Cell. Immunol. **161**: 181–187.

Tesch H., Hohlfeld R., Toyka K.V. (1989). Analysis of immunoglobulin and T cell receptor gene rearrangements in the thymus of myasthenia gravis patients. J. Neuroimmunol. **21**: 169–176.

Truffault F., Cohen Kaminsky S., Khalil I., Levasseur P., Berrih Aknin S. (1997). Altered intrathymic T-cell repertoire in human myasthenia gravis. Ann. Neurol. **41**: 731–741.

van Bergen J. and Koning F. (1998). Altered peptide ligands and wild-type peptide induce indistinguishable responses of a human Th0 clone. Eur. J. Immunol. **28**: 2801–2808.

van der Kwast T.H., van Vliet E., Cristen E., van Ewijk W., van der Heul R.O. (1985). An immunohistologic study of the epithelial and lymphoid components of six thymomas. Hum. Pathol. **16**: 1001–1008.

van Eggermond M.C., Rijkers G.T., Kuis W., Zegers B.J., van den Elsen P.J. (1993). T cell development in a major histocompatibility complex class II-deficient patient. Eur. J. Immunol. **23**: 2585–2591.

Vanhecke D., Verhasselt B., De Smedt M., De Paepe B., Leclercq G., Plum J., Vandekerckhove B. (1997). MHC class II molecules are required for initiation of positive selection but not during terminal differentiation of human CD4 single positive thymocytes. J. Immunol. **158**: 3730–3737.

Vegh Z., Wang P., Vanky F., Klein. E. (1993). Selectively down-regulated expression of major histocompatibility complex class I alleles in human solid tumors. Cancer Res. **53** (10 Suppl): 2416–20.

Vignali D.A. and Strominger J.L. (1994). Amino acid residues that flank core peptide epitopes and the extracellular domain of CD4 modulate differential signaling through the T cell receptor. J. Exp. Med. **179**: 1945–1956.

von Gaudecker B., Steinmann G.G., Hansmann M.L., Harpprecht J., Milicevic N.M., Müller-Hermelink H.K. (1986) Immunohistochemical characterization of the thymic microenvironment. A light-microscopic and ultrastructural immunocytochemical study. Cell Tissue Res. **244**: 403–12.

Wilisch A., Schultz A., Jung A., Schalke B., Toyka K.V., Pallini V., Müller-Hermelink H.K. and Marx A. (1997). Titin epitope in thymoma. In: Epithelial Tumors of the Thymus (Pathology, Biology, Treatment), Marx A. and Müller-Hermelink H.K., eds. (London, Plenum Press), pp. 221–227.

Willcox N., Schluep M., Ritter M.A., Schuurman H.J., Newsom Davis J., Christensson B. (1987). Myasthenic and nonmyasthenic thymoma: an expansion of a minor cortical epithelial cell subset? Am. J. Pathol. **127**: 447–460.

Williams O., Tanaka Y., Tarazona R and Kioussis D. (1997). The agonist-antagonist balance in positive selection. Immunol. Today **18**: 121–126;.

Zuniga-Pflücker J.C., McCarthy S.A., Weston M., Longo D.L., Singer A., Kruisbeek A.M. (1989). Role of CD4 in thymocyte selection and maturation. J. Exp. Med. **169**: 2085–2096.

